# LinkageMapView—rendering high-resolution linkage and QTL maps

**DOI:** 10.1093/bioinformatics/btx576

**Published:** 2017-09-13

**Authors:** Lisa A Ouellette, Robert W Reid, Steven G Blanchard, Cory R Brouwer

**Affiliations:** Department of Bioinformatics and Genomics, University of North Carolina Charlotte, Kannapolis, NC, USA

## Abstract

**Motivation:**

Linkage and quantitative trait loci (QTL) maps are critical tools for the study of the genetic basis of complex traits. With the advances in sequencing technology over the past decade, linkage map densities have been increasing dramatically, while the visualization tools have not kept pace. LinkageMapView is a free add-on package written in R that produces high resolution, publication-ready visualizations of linkage and QTL maps. While there is software available to generate linkage map graphics, none are freely available, produce publication quality figures, are open source and can run on all platforms. LinkageMapView can be integrated into map building pipelines as it seamlessly incorporates output from R/qtl and also accepts simple text or comma delimited files. There are numerous options within the package to build highly customizable maps, allow for linkage group comparisons, and annotate QTL regions.

**Availability and implementation:**

https://cran.r-project.org/web/packages/LinkageMapView/

## 1 Introduction

Linkage studies over the years have been producing genetic maps (linkage maps) that act as invaluable tools in areas of genetic disease detection, anchoring genome sequence assemblies and elucidating genetic mechanisms of agronomical important traits in plants. The advent of high-throughput sequencing has allowed for better marker identification and greater map densities. This is a desirable outcome for solving many genetics based problems but visualizing these maps creates a new challenge. As the marker density increases, it becomes difficult to add labels, QTL regions and centimorgan values and still maintain readability.

There is existing software that generates linkage and QTL maps and these fall into three categories. Rudimentary maps are available in software where the primary objective is QTL analysis. For example, R/qtl (Broman *et al.*, 2003) is widely used for QTL analysis but the plot functionality is not a strength. High-quality maps can be produced with feature-rich software such as MapChart (Voorrips, 2002). MapChart only runs on Windows. Lastly, there is web-based software like R/qtlcharts (Broman, 2015), but it is more useful for interactively exploring linkage and QTL maps. Here, we introduce LinkageMapView, an open source package in R that can produce publication quality genetic maps, can be run on any platform supported by R, and can be incorporated into a user’s existing R-based pipeline.

## 2 Software

LinkageMapView uses R base graphics for plotting, labels and optional colored segments. The main function, lmv.linkage.plot, is the only function a user needs to directly invoke. The generated maps are output in Adobe Portable Document Format (PDF). Because linkage maps can be dense with loci, LinkageMapView has many options that allow the user to reduce extraneous detail, minimize map dimensions, and highlight areas of importance.

## 3 Program overview

### 3.1 Program input data

Input to LinkageMapView is a delimited text file with the first three columns containing linkage group name, cM position, and locus. Alternatively, input can be an R/qtl (Broman *et al.*, 2003) cross object.

### 3.2 Program usage

To generate the map, a user calls the lmv.linkage.plot function. Parameters allow users to customize most aspects of the map. Users can alter map size, title, fonts, colors and label display to emphasize markers or QTLs of importance. Some common parameters are discussed below.

mapthese: An ordered vector of linkage group names to print.

showonly: A vector of loci labels to print. All other loci labels will not be printed although their presence is depicted with a line segment across the chromosome to indicate loci density.

dupnbr: If TRUE, the first locus name at a position will be shown followed by a count of the duplicate markers at that position.

ruler: If TRUE, instead of printing position numbers on each chromosome, a ruler is printed on the left side.

markerformatlist: A list of loci with the desired R font, color, and size for the labels. This list overrides any other specifications for the loci listed.

qtldf: An optional R data frame containing QTL information:

autoconnadj: If TRUE (the default), loci with the same name and in adjacent linkage groups will be connected with a line.

conndf: An optional data frame containing the homologs to connect with a line. If autoconnadj is TRUE, this list will be merged with the automatic list.

revthese: An optional vector of linkage group names to reverse. The end position becomes position 0 and position 0 becomes the largest position.

posonleft: An optional boolean vector the length of the number linkage groups to map, indicating if the positions should be plotted on the left (TRUE) or on the right (FALSE).

## 4 Results

**Fig. 1:**
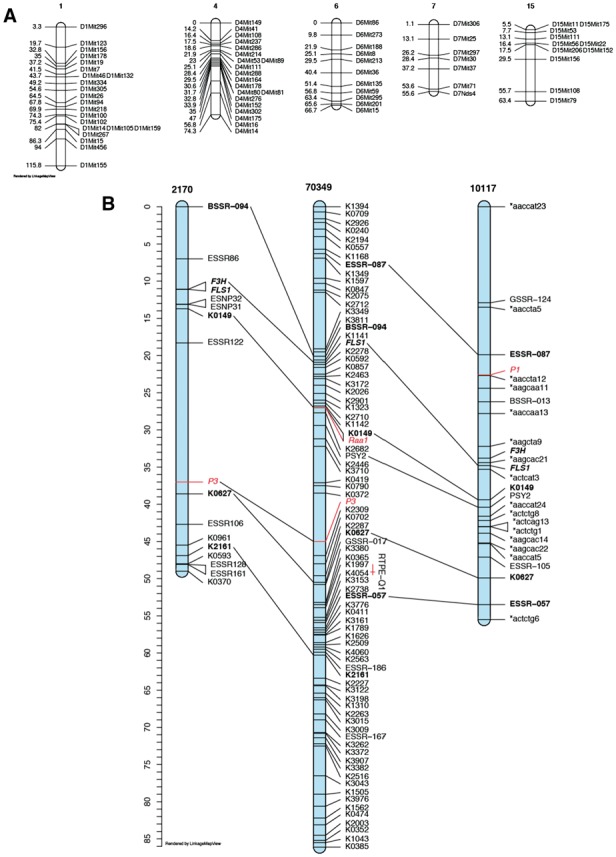
(**A**) Map from R/qtl (Broman *et al.*, 2003) cross object using the hyper sample data from the R/qtl package. (**B**) A comparative map of three populations of carrot (Cavagnaro *et al.*, 2011)

The maps from two use cases are shown in Figure 1.

The first case (Fig. 1A) demonstrates using a cross object from R/qtl as input to LinkageMapView (Listing 1) using all default formats.

**Listing 1.** Sample commands to generate linkage group map from R/qtl cross object.

library(qtl)

data(hyper)

lmv.linkage.plot(hyper,"qtlhyper.pdf", mapthese = c(1, 4, 6, 7, 15))

The second case (Fig. 1B) demonstrates using LinkageMapView to produce a comparative map with homologs connected, a QTL mapped, a cM ruler and several color and font options.

## 5 Conclusion

LinkageMapView is a freely available and open source tool to produce publication quality linkage and QTL maps. It is implemented in R to provide easy integration with R QTL analysis programs and so it can be run on any platform. The plethora of options provides the user with flexibility in dealing with high**-**density linkage maps. These characteristics make LinkageMapView more useful than currently available linkage map plotting tools.
